# Case Report: Secondary syphilis with pulmonary involvement mimicking eosinophilic granulomatosis with polyangiitis — targeted next-generation sequencing as an adjunct in resolving an atypical non-exanthematous presentation

**DOI:** 10.3389/fmed.2026.1874185

**Published:** 2026-09-03

**Authors:** Shunjun Tang, Tiehan Zhang, Aiyi Hao, Bin Su, Bin Liu

**Affiliations:** 1 Department of Infectious Diseases, Characteristic Medical Center of Chinese People’s Armed Police Force, Tianjin, China; 2 Integrative Medical Center, Tianjin University, Tianjin, China

**Keywords:** case report, eosinophilia, pulmonary syphilis, targeted next-generation sequencing, Treponema pallidum

## Abstract

**Background:**

The global resurgence of syphilis poses growing challenges for clinical diagnosis, particularly when atypical presentations without classic mucocutaneous lesions mimic systemic autoimmune diseases. Pulmonary involvement in secondary syphilis is rare and frequently misdiagnosed, leading to inappropriate immunosuppressive therapy.

**Case presentation:**

A 24-year-old male presented with one month of inflammatory back pain and two weeks of cough, purulent sputum, and intermittent low-grade fever. He initially denied high-risk sexual exposure. Physical examination revealed no rash, chancre, or lymphadenopathy. Laboratory workup showed marked eosinophilia (15.70%), elevated C-reactive protein, and immunoglobulin E (1,290 IU/mL). Chest computed tomography demonstrated bilateral patchy infiltrates and multiple small upper-lobe nodules. While this presentation suggested eosinophilic granulomatosis with polyangiitis (EGPA), unexpected positive syphilis serology (rapid plasma reagin 1:128) redirected the diagnostic workup. Upon further confidential questioning, the patient acknowledged high-risk sexual exposures. Bronchoalveolar lavage fluid targeted next-generation sequencing (tNGS) detected a high load of Treponema pallidum (11,732 reads) alongside co-infecting respiratory pathogens. EGPA was rendered highly unlikely by complete clinical and radiological resolution following antimicrobial therapy alone, without corticosteroids.

**Conclusion:**

This case illustrates that non-rash secondary pulmonary syphilis can closely mimic EGPA, and that tNGS provides a valuable adjunctive tool for resolving such diagnostic challenges, thereby preventing potentially harmful immunosuppressive treatment. As syphilis continues to re-emerge globally, clinicians should maintain a high index of suspicion for syphilitic pulmonary involvement in patients with unexplained eosinophilia and pulmonary infiltrates, even in the absence of dermatological manifestations.

## 1 Introduction

Syphilis, caused by the spirochete *Treponema pallidum*, has undergone a dramatic global resurgence over the past decade, with incidence rates rising across all continents and demographic groups (1, 2). This re-emergence has re-established syphilis as a major public health priority requiring enhanced surveillance, prevention, and treatment strategies. The infection is classically known as “the great imitator” for its protean clinical manifestations, yet secondary syphilis is still predominantly recognized by its characteristic mucocutaneous rash. Pulmonary involvement in secondary syphilis remains rare and poorly characterized, with fewer than 30 cases documented in the English-language literature (3–5).

The absence of dermatological manifestations in pulmonary involvement of syphilis creates a critical diagnostic blind spot. The coexistence of pulmonary nodules and peripheral eosinophilia often raises clinical suspicion for eosinophilic lung diseases, including eosinophilic granulomatosis with polyangiitis (EGPA), creating a potential diagnostic trap (6). Misdiagnosis in this context carries severe consequences: corticosteroids and cytotoxic agents administered for presumed EGPA would permit unchecked treponemal dissemination and potentially fatal neurological or cardiovascular complications. From a surveillance perspective, every unrecognized case represents a missed opportunity for partner notification and interruption of community transmission.

Targeted next-generation sequencing (tNGS) of bronchoalveolar lavage fluid (BALF) has emerged as a valuable adjunctive diagnostic tool for infectious diseases, capable of detecting a broad spectrum of pathogens with high sensitivity and specificity (7). Its application in resolving the differential diagnosis between infectious and non-infectious pulmonary syndromes is of particular value when conventional microbiological investigations remain negative. Detailed tNGS methodology is provided in Supplementary Methods S1.

Here, we present a case of non-rash secondary pulmonary syphilis presenting with marked eosinophilia, where tNGS played a key role in distinguishing an infectious mimic from EGPA, enabling timely targeted antimicrobial therapy and averting inappropriate immunosuppression. We discuss the implications of this case for syphilis surveillance strategies, prevention of diagnostic errors, and treatment considerations.

## 2 Case description

A 24-year-old male presented to the respiratory outpatient clinic with a two-week history of paroxysmal cough productive of purulent sputum (initially white, progressing to yellow) and intermittent low-grade fever (maximum temperature <38 degrees C) (Table 1). He also reported a one-month history of inflammatory back and bilateral flank pain characterized by morning exacerbation and improvement with physical activity. This pain had been treated with a one-month course of oral celecoxib without relief. He had a two-year history of gout not on regular treatment, longstanding allergic rhinitis exacerbated by cat dander exposure, and a one-year smoking history.

**Table 1:** Timeline of Key clinical events.

Timepoint	Clinical Presentation	Key Diagnostics	Interventions
Day −30	Inflammatory back & flank pain	x-ray: No obvious abnormalities	Oral celecoxib (no relief)
Day −14	Onset of paroxysmal cough, purulent sputum, and intermittent low-grade fever	—	—
Day 0	Outpatient presentation with persistent respiratory and musculoskeletal symptoms	WBC 11.04 × 10^9^/L, Eos 15.70% (absolute 1.73 × 10^9^/L), CRP 42.68 mg/L;	Intravenous nemonoxacin malate started empirically
		Chest CT: bilateral patchy infiltrates & upper lobe nodules	
Day 1	Admission. Afebrile, stable vital signs. No skin rash, chancre, or lymphadenopathy	Comprehensive infectious, immune, and autoimmune workup (including ANA, ANCA, MTB/RIF) ordered	—
Day 2	No dermatological manifestations observed.	TPPA (+) (RPR pending);	BAL performed for tNGS
	Patient acknowledged high-risk sexual exposures	D-dimer 670.00 ng/mL, ESR 76 mm/h, IL-6 67.32 pg/mL, CD4/CD8 1.10	
Day 3	Respiratory symptoms persist	BALF tNGS: *T. pallidum* (11,732 reads), Rhinovirus (767,068 reads), EBV (66,215 reads), *S. pneumoniae* (493 reads)	Nemonoxacin discontinued;
			IV ceftriaxone (2 g/day) initiated (neurosyphilis dosage)
Day 5	—	RPR 1:128; IgE 1,290 IU/mL, C3 0.71 g/L, C4 0.087 g/L, Anticardiolipin antibody (+);	—
		ANCA panel (including anti-PR3 and anti-MPO antibodies) (−)	
Day 15	Fever, cough, and back pain resolved. Ready for discharge	Eos 18.10% (absolute peak 2.12 × 10^9^/L), RPR 1:128	Transition to IM benzathine penicillin G (3 weekly doses)
Day 30	Sustained clinical improvement	RPR 1:32	—
Day 60	—	RPR 1:8	—
Day 100	Asymptomatic, returned to baseline functional status	RPR 1:2;	Case reported to registry; partner notification advised
		Chest CT: near-complete resolution of infiltrates	

ANA, antinuclear antibody; ANCA, antineutrophil cytoplasmic antibody; BAL, bronchoalveolar lavage; BALF, bronchoalveolar lavage fluid; CRP, C-reactive protein; CT, computed tomography; EBV, Epstein–Barr virus; Eos, eosinophil; ESR, erythrocyte sedimentation rate; IFN, interferon; IgE, immunoglobulin E; IgG, immunoglobulin G; IL, interleukin; IM, intramuscular; MPO, myeloperoxidase; MTB/RIF, *Mycobacterium tuberculosis*/rifampin resistance; MU, million units; PR3, proteinase 3; RPR, rapid plasma reagin; tNGS, targeted next-generation sequencing; TPPA, *Treponema pallidum* particle agglutination assay; WBC, white blood cell.

**Figure 1:**
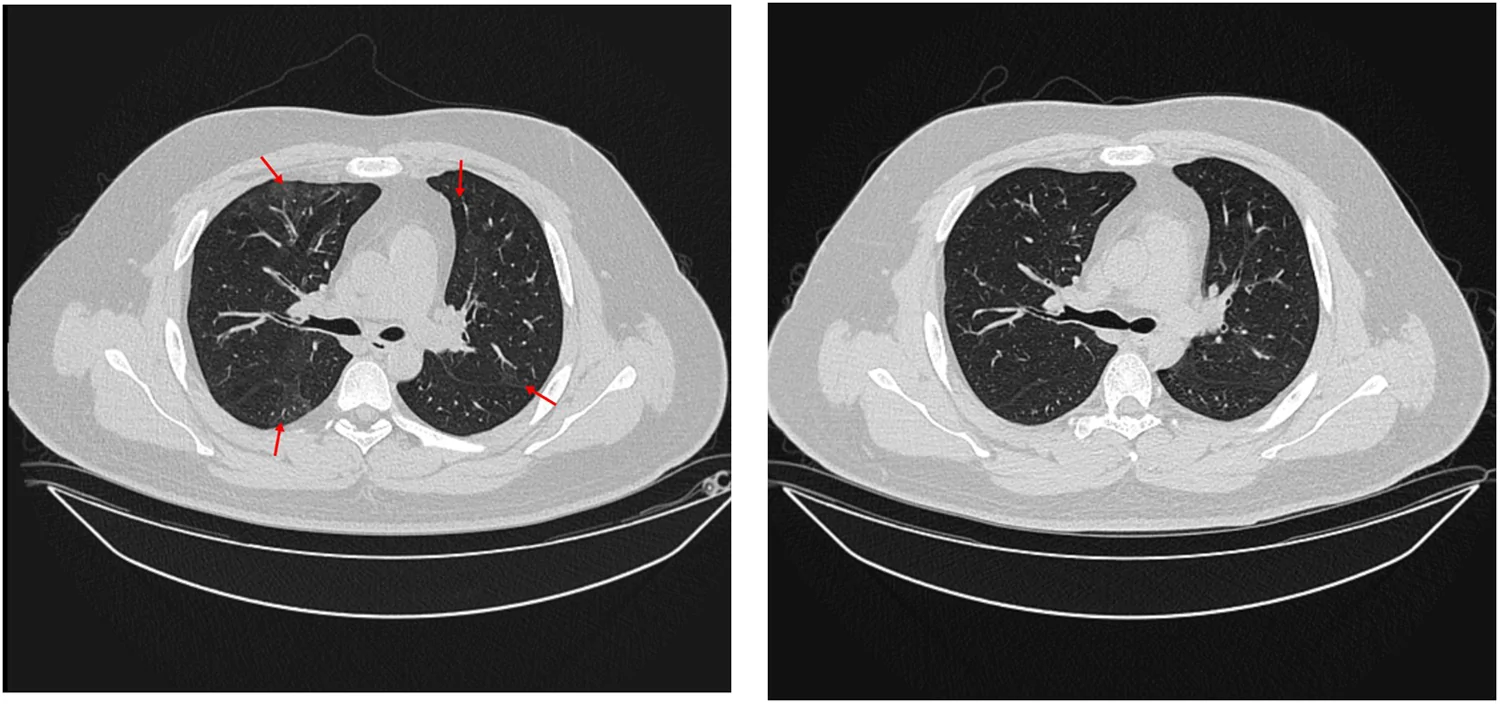
Chest computed tomography (CT) imaging. (Left) Initial CT on admission (Day 0) reveals bilateral multifocal patchy ground-glass and consolidative opacities (red arrows), accompanied by multiple small nodules in the upper lobes (identified on formal radiology department interpretation; below visual resolution on standard-view screenshots). (Right) Follow-up CT at 100 days demonstrates near-complete resolution of the bilateral patchy infiltrates, whereas the upper lobe nodules remain largely unchanged (per formal radiology department report).

Initial laboratory investigations on the day of presentation (Day 0) revealed leukocytosis with marked eosinophilia and elevated C-reactive protein (Table 2). Chest computed tomography (CT) (Figure 1, left) revealed bilateral multifocal patchy ground-glass and consolidative opacities, as well as multiple small nodules in both upper lobes.

**Table 2:** Summary of Key diagnostic findings (Day 0 to Day 3).

Category	Investigation	Result	Reference Range
Routine Blood & Inflammatory Markers	White Blood Cell (WBC) count	11.04 × 10^9^/L	3.50–9.50 × 10^9^/L
	Eosinophil percentage (Eos%)	15.70% (Peak: 18.10%)	0.40%–8.00%
	Absolute eosinophil count	1.73 × 10^9^/L (Peak: 2.12 × 10^9^/L)	0.02–0.52 × 10^9^/L
	C-reactive protein (CRP)	42.68 mg/L	0.00–10.00 mg/L
	Erythrocyte sedimentation rate (ESR)	76 mm/h	0–15 mm/h
	D-dimer (plasma)	670.00 ng/mL	0.00–500.00 ng/mL
	Fibrinogen	7.67 g/L	2.00–4.00 g/L
Immunological & Autoimmune Profile	Total IgE	1,290 IU/mL	0.0–100.0 IU/mL
	IgG	18.60 g/L	7.00–16.00 g/L
	Interleukin-6 (IL-6)	67.32 pg/mL	<18.60 pg/mL
	IL-4/IFN-*γ*	Normal	Within normal limits
	CD4/CD8 ratio	1.10	1.4–2.0
	Complement C3	0.71 g/L	0.90–1.80 g/L
	Complement C4	0.087 g/L	0.100–0.400 g/L
	Anticardiolipin antibody	Positive (+)	Negative
Syphilis Serology	TPPA	Positive (+)	Negative
	RPR titer	1:128	Non-reactive (Negative)
BALF Cytology	Nucleated cell count	130 × 10^6^/L	—
	Neutrophils	29%	0%–2%
	Lymphocytes	10%	0%–12%
	Macrophages	61%	—
BALF tNGS Detection	*T. pallidum* reads	11,732	Negative
	Rhinovirus reads	767,068	Negative
	EBV reads	66,215	Negative
	*S. pneumoniae* reads	493	Negative
Key Exclusionary Tests	ANCA panel (including PR3 & MPO)	Negative	Negative
	Antinuclear antibody (ANA)	Negative	Negative
	T-SPOT.TB/MTB/RIF	Negative	Negative
	Sputum bacterial/fungal culture	Negative	Negative

ANA, antinuclear antibody; ANCA, antineutrophil cytoplasmic antibody; BAL, bronchoalveolar lavage; BALF, bronchoalveolar lavage fluid; CRP, C-reactive protein; CT, computed tomography; EBV, Epstein–Barr virus; Eos, eosinophil; ESR, erythrocyte sedimentation rate; IFN, interferon; IgE, immunoglobulin E; IgG, immunoglobulin G; IL, interleukin; IM, intramuscular; MPO, myeloperoxidase; MTB/RIF, *Mycobacterium tuberculosis*/rifampin resistance; MU, million units; PR3, proteinase 3; RPR, rapid plasma reagin; tNGS, targeted next-generation sequencing; TPPA, *Treponema pallidum* particle agglutination assay; WBC, white blood cell.

On admission (Day 1), the patient's vital signs were: temperature 37.7°C, heart rate 98 bpm, respiratory rate 25/min, blood pressure 154/98 mmHg. Physical examination revealed no rash — including on the palms and soles — no oral, pharyngeal, or genital mucosal lesions, and no palpable superficial lymphadenopathy. He initially denied any history of unprotected sexual contact. The preliminary differential diagnosis prioritized EGPA, parasitic infection, fungal infection, and tuberculosis. A comprehensive diagnostic workup was initiated (summarized in Table 2). Empirical intravenous nemonoxacin malate was commenced pending results. Bronchoscopy with BALF was scheduled for the following day.

**Figure 2:**
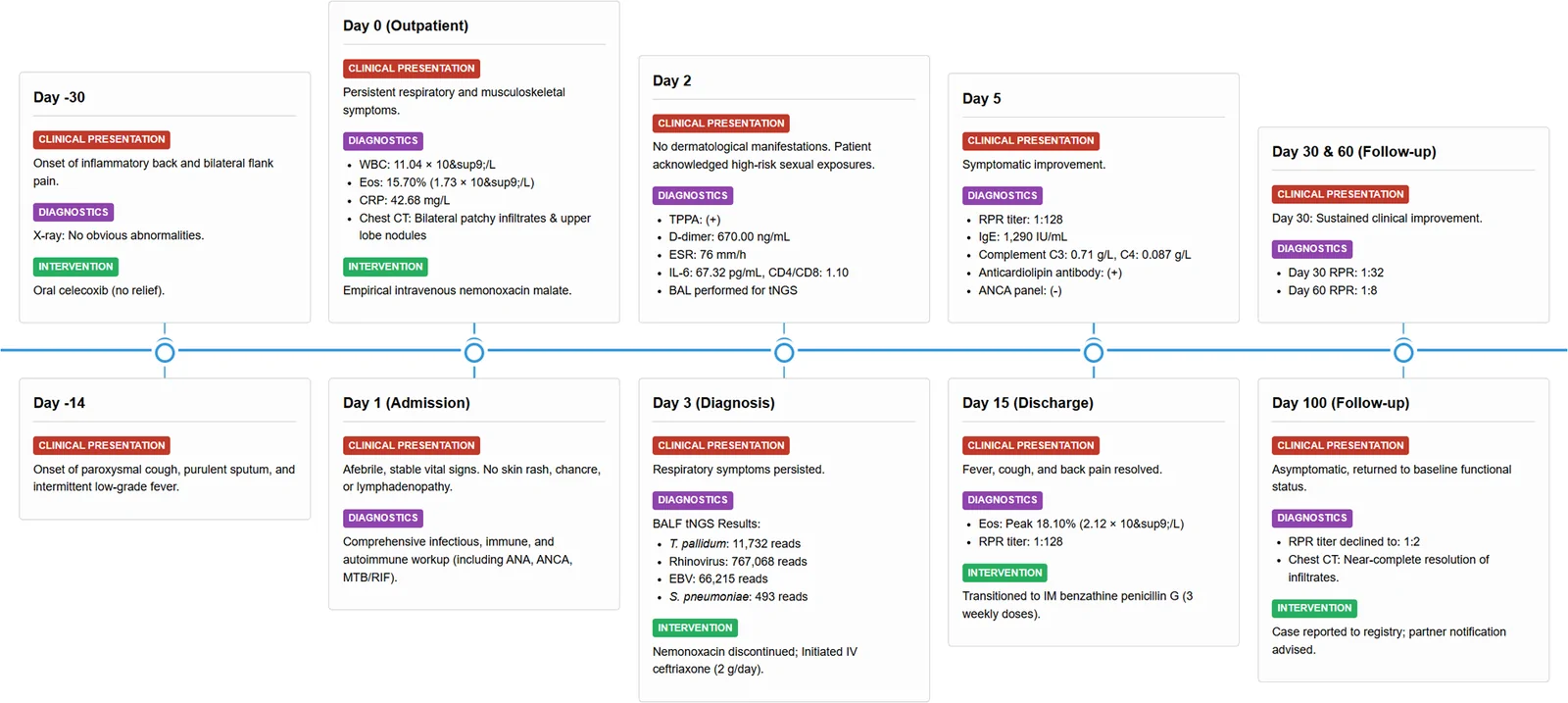
Clinical course timeline. The timeline summarizes key clinical presentations, diagnostic findings, and therapeutic interventions from symptom onset (Day −30) through final follow-up (Day 100). BAL, bronchoalveolar lavage; BALF, bronchoalveolar lavage fluid; CRP, C-reactive protein; CT, computed tomography; EBV, Epstein–Barr virus; Eos, eosinophil; ESR, erythrocyte sedimentation rate; IL-6, interleukin-6; IM, intramuscular; IV, intravenous; MTB/RIF, Mycobacterium tuberculosis/rifampin resistance; RPR, rapid plasma reagin; tNGS, targeted next-generation sequencing; TPPA, *Treponema pallidum* particle agglutination assay; WBC, white blood cell.

On Day 2, routine pre-bronchoscopy serological screening unexpectedly returned a positive *T. pallidum* particle agglutination assay (TPPA) and a reactive RPR test (1:128). Upon further confidential questioning, the patient acknowledged high-risk sexual exposures. BAL was performed in the right upper lobe; the bronchoscopic appearance showed no focal mucosal abnormalities. BALF was submitted for tNGS and routine cytology. The complete clinical course is summarized in Figure 2.

## 3 Diagnostic assessment

The BALF tNGS results, returned on Day 3, provided important supporting evidence for the diagnosis. The assay detected a high read count of *T. pallidum* subspecies *pallidum* (11,732 sequence reads). Concurrent pathogens identified included *S. pneumoniae*, rhinovirus, and Epstein–Barr virus (EBV). Although the patient's presentation — pulmonary infiltrates, marked eosinophilia, and multi-system involvement — closely mimicked EGPA, the 2022 ACR/EULAR classification criteria for EGPA explicitly require exclusion of infection mimics before their application (6). Subsequent immunological profiling (summarized in Table 2) revealed negative ANCA, elevated IgG, a distinctive cytokine dissociation (marked elevation of IL-6 with normal Th2 cytokines IL-4 and IFN-*γ*), complement depletion, and CD4/CD8 inversion — a profile more consistent with infection-driven immune dysregulation than with primary autoimmune disease. Additional exclusionary testing — including T-SPOT.TB, sputum bacterial and fungal cultures, and ANA — were negative (Table 2). BALF cytology demonstrated neutrophilic predominance, consistent with an infectious inflammatory process rather than an eosinophilic lung disease. Taken together, the tNGS findings, immunological abnormalities, clinical presentation, and radiological features identified secondary pulmonary syphilis with multi-pathogen co-infection as the primary etiology, though histopathological confirmation was not obtained.

## 4 Therapeutic intervention

Upon diagnosis, empirical nemonoxacin was discontinued. Benzathine penicillin G, the standard first-line therapy for syphilis, was temporarily unavailable. Given the inability to clinically exclude neurosyphilis—lumbar puncture was recommended but declined by the patient—intravenous ceftriaxone 2 g once daily was initiated as the recommended alternative regimen with adequate central nervous system penetration (8, 9). Ceftriaxone also provided coverage for the co-detected *S. pneumoniae*. After a two-week course, the patient's fever, cough, sputum production, and back pain had improved substantially. Once benzathine penicillin G became available, three weekly doses of intramuscular benzathine penicillin G (2.4 million units) were administered to complete the standard treatment course (8, 9). This sequential approach—ceftriaxone bridging followed by guideline-directed benzathine penicillin—was adopted because the two-week ceftriaxone course alone was considered insufficient for possible late latent or neurosyphilis; the addition of benzathine penicillin ensured sustained treponemicidal coverage over the recommended treatment duration while navigating the initial unavailability of first-line therapy. The patient was counseled regarding partner notification and safer sexual practices (10).

## 5 Follow-up and outcomes

At two weeks after initiation of ceftriaxone therapy (Day 15), peripheral eosinophilia paradoxically increased to 18.10% before subsequently declining. At the 100-day follow-up, the RPR titer had declined significantly to 1:2. Repeat chest CT demonstrated near-complete resolution of the previously noted bilateral patchy infiltrates. The multiple small upper-lobe nodules remained largely unchanged, consistent with the formal radiology department interpretation, although their precise etiology—syphilitic granulomas vs. pre-existing lesions—could not be definitively determined. Longer-term follow-up imaging is planned (Figure 1, right). Clinically, the patient had returned to his baseline functional status. The concordant clinical, serological, and radiological response to anti-treponemal therapy — achieved without corticosteroid administration — provided *post-hoc* confirmation of the infectious etiology. The case was reported to the local infectious disease registry, and the patient was counseled to notify his partner for screening.

## 6 Discussion

While occasional cases of syphilis-related peripheral or tissue eosinophilia have been reported (11, 12), presentation with marked eosinophilia mimicking systemic vasculitis remains exceptionally rare.

The marked peripheral eosinophilia (peak 2.12 × 10^9^/L) warranted a systematic differential diagnosis. A drug reaction was considered unlikely: eosinophilia preceded antibiotic administration, and the patient had been on a stable dose of celecoxib without new drug exposure. Parasitic infection was not supported by negative stool routine examination or relevant epidemiological history, though dedicated serological testing was not performed. Fungal infection was excluded by negative sputum fungal culture, galactomannan testing, and tNGS. Allergic disease alone could not account for the magnitude of eosinophilia (2.12 × 10^9^/L) or the multisystem involvement. Hematological disorders were inconsistent with the isolated eosinophil elevation and absence of other cell-line abnormalities. Eosinophilic pneumonia could not be definitively excluded without BALF differential cell count; however, complete resolution without corticosteroid therapy would be atypical for this condition.

The complete absence of mucocutaneous lesions throughout the disease course is particularly notable and challenges the conventional clinical case definition used in many syphilis surveillance systems, which often rely on visible skin findings as sentinel diagnostic clues (13). As syphilis incidence continues to climb globally — including in populations not traditionally considered at high risk—the proportion of cases presenting with atypical or organ-specific manifestations is likely to increase. This case suggests that molecular diagnostic data, such as tNGS-detected *T. pallidum*, may complement existing surveillance approaches for pulmonary syndromes of unknown etiology. From a public health standpoint, every missed or delayed diagnosis of infectious syphilis represents not only a threat to the individual patient but also a missed opportunity for interrupting community transmission through timely partner notification (14).

Syphilis-associated eosinophilia is exceedingly rare (11, 12). Rhinovirus (767,068 reads) was considered an active co-infection; while it may have amplified local ILC2-dependent pulmonary eosinophilia (15), it cannot account for the systemic EGPA-like presentation (peak eosinophilia 2.12 × 10^9^/L, IgE 1,290 IU/mL), which was primarily driven by secondary syphilis (16, 17). *Streptococcus pneumoniae* (493 reads) likely represented a secondary bacterial infection accounting for the purulent sputum, and EBV (66,215 reads) reflected latent reactivation under immune perturbation. Furthermore, the high *T. pallidum* read count and concordant radiographic resolution argue against incidental bronchoscopic carryover from upper airway mucosal colonization.

To our knowledge, this represents the first application of tNGS in diagnosing pulmonary syphilis; the only prior NGS-based report in this setting employed metagenomic sequencing (4). Unlike shotgun mNGS, tNGS uses targeted multiplex PCR enrichment, which achieves higher sensitivity for low-abundance organisms and lower host background. These characteristics likely facilitated the unambiguous detection of *T. pallidum* in a BALF specimen co-infected with high-load rhinovirus, and support the consideration of tNGS as a practical diagnostic option for rare pulmonary infections when invasive biopsy is infeasible.

The cytokine profile—elevated IL-6 with normal IL-4 and IFN-*γ*—is compatible with local ILC2-driven eosinophilia rather than systemic Th2 polarization, though this distinction cannot be made on the basis of cytokine data alone. We hypothesize that *T. pallidum* infection, rhinovirus-induced alarmin release, and the patient's atopic background may have synergistically amplified the eosinophilic response, and the post-treatment eosinophil peak could reflect ILC2-IL-5 axis inertia (18). However, this proposed mechanism remains speculative: IL-5, ILC2 activation, and local type 2 immune responses were not directly measured. The value of this model lies in generating testable hypotheses for future studies rather than providing a confirmed mechanistic explanation.

The temporary unavailability of intravenous penicillin at the time of diagnosis necessitated the use of ceftriaxone, a recommended alternative for the treatment of syphilis (8, 9). The favorable clinical and serological response observed in this case adds to the growing body of real-world evidence supporting the efficacy of ceftriaxone in the management of syphilis, including cases with potential neurological involvement. However, the global reliance on benzathine penicillin as first-line therapy renders syphilis treatment vulnerable to supply-chain disruptions — an important consideration for treatment guideline development and antimicrobial stewardship policy (19). The sequential approach employed here (intravenous ceftriaxone followed by intramuscular benzathine penicillin) ensured adequate treatment duration and tissue penetration while navigating temporary drug unavailability.

We acknowledge certain limitations in this case report. First, because of the rapid clinical improvement following antibiotic therapy, a transbronchial or percutaneous lung biopsy was not performed. Consequently, definitive histopathological confirmation demonstrating treponemal invasion within the granulomatous nodules was not obtained. Nevertheless, the detection of *T. pallidum* at high read counts in BALF by tNGS, together with the consistent serological evidence and the complete clinical and serological response to anti-treponemal therapy, provides a level of diagnostic confidence sufficient to guide clinical management.

## 7 Patient perspective

The patient reported significant psychological distress during the diagnostic process, particularly when confronted with the possibility of a systemic vasculitis requiring long-term immunosuppression. He expressed relief upon receiving a definitive infectious diagnosis with a clear, time-limited treatment course. He acknowledged that the stigma associated with sexually transmitted infections had contributed to his initial withholding of sexual history, and he emphasized the importance of non-judgmental, repeated sexual history-taking in clinical practice. The patient was satisfied with his clinical outcome.

## 8 Conclusion

This case of non-rash secondary pulmonary syphilis presenting with marked eosinophilia and masquerading as EGPA highlights three interconnected lessons for the fields of surveillance, prevention, and treatment of infectious diseases. First, the absence of dermatological findings should not dissuade clinicians from considering syphilis in the differential diagnosis of unexplained pulmonary syndromes, particularly in the context of the ongoing global resurgence. Second, tNGS represents a valuable adjunctive diagnostic tool capable of simultaneously identifying the causative pathogen and excluding non-infectious mimics, thereby preventing potentially harmful misdiagnosis and inappropriate immunosuppression. Third, ceftriaxone provides an effective alternative to penicillin when the latter is unavailable, though health systems should strengthen supply-chain resilience for first-line antitreponemal therapies. As syphilis continues to re-emerge as a global public health threat, heightened clinical awareness, integration of advanced molecular diagnostics, and robust treatment pathways are essential components of an effective response.

## Data Availability

The original contributions presented in the study are included in the article/Supplementary Material, further inquiries can be directed to the corresponding author/s.
