# Supplementary material for: Case Report: Secondary syphilis with pulmonary involvement mimicking eosinophilic granulomatosis with polyangiitis — targeted next-generation sequencing as an adjunct in resolving an atypical non-exanthematous presentation
Source: Front Med (Lausanne). 2026 Sep 3;13:1874185. doi: 10.3389/fmed.2026.1874185 (PMC13581970; doi:10.3389/fmed.2026.1874185)
Supplement: Supplementary file 1 [file Table1.docx]

**Supplementary Methods S1: Targeted Next-Generation Sequencing (tNGS) Methodology**(1)

**S1.1 Sample Collection and Nucleic Acid Extraction**

Bronchoalveolar lavage fluid (BALF) samples (3–5 mL) were collected into sterile tubes following standard clinical collection procedures and transported at 4–8 °C. Nucleic acid extraction was performed using the PathoXtract® Universal Nucleic Acid Extraction Kit (magnetic bead method)	(WYXM10002D, Willingmed, Beijing, China)	according	to	the	manufacturer's instructions. The kit simultaneously extracts DNA and RNA from clinical specimens.

**S1.2 Library Preparation and Sequencing**

cDNA synthesis, targeted multiplex PCR pre-amplification of pathogen-specific genomic regions, and library preparation were carried out using the PathoLib™ Pathogen Targeted Detection Kit (WYLM10001S, Willingmed, Beijing, China). The resulting libraries were quantified using a Qubit 4.0 Fluorometer (Thermo Fisher Scientific, Waltham, MA, USA) to ensure that the library concentration of all samples was ≥ 0.5 ng/μL. Sequencing was performed on the MGISEQ-200 platform (MGI Tech, Shenzhen, China) using a 50-bp sequencing strategy, and each library was sequenced to a minimum of 0.1 million (0.1 M) reads.

**S1.3 Bioinformatics Analysis**

Raw sequencing data were processed with Fastp v0.20.1 for adapter trimming and low-quality base filtering using default parameters. Clean reads were aligned to a reference database using BWA (Burrows-Wheeler Aligner) to obtain species identity and abundance information. The reference sequences in the	custom database were	derived from multiple publicly	available resources, including the RefSeq database and the NCBI Nucleotide database. The Comprehensive Antibiotic Resistance Database (CARD), the Virulence Factor Database (VFDB), and relevant literature were used as references for the annotation of antimicrobial resistance genes and virulence genes. The assay reports the raw sequence read count for each detected microorganism as a semi-quantitative indicator of organism burden.

**S1.4 Quality Control**

Each batch of samples included the following: (i) a positive control to verify the performance of the entire workflow from nucleic acid extraction through sequencing; (ii) a negative control

(nuclease-free water) processed and run alongside to monitor for contamination throughout the entire tNGS workflow; (iii) an internal reference to confirm adequate sample processing and amplification; and (iv) sequencing data volume monitoring to ensure a minimum threshold of 0.1 M reads. All quality control indicators (positive control, negative control, internal reference, and sequencing data volume) were required to pass pre-specified acceptance criteria before data release.

**S1.5 Detection Panel Characteristics**

The Ampan™ Respi targeted next-generation sequencing assay (Willingmed, Beijing, China) is a commercially available, NMPA-registered *in vitro* diagnostic product designed for the detection of respiratory tract infection-associated pathogens. The panel employs a targeted multiplex PCR to amplify pathogen-specific genomic regions, enabling the simultaneous detection of bacteria (including *Treponema pallidum*, *Streptococcus pneumoniae*, and other clinically relevant bacterial species), DNA and RNA viruses (via reverse transcription), fungi, and parasites. The panel also includes targets for selected antimicrobial resistance genes and virulence genes. Specific details regarding the number of independent target loci, coverage and depth, and precise target genomic regions are proprietary information held by the manufacturer.

**S1.6 Interpretation Criteria and Limitations**

Detection results were interpreted based on the presence and magnitude of pathogen-specific sequence reads, with the raw read count serving as a semi-quantitative indicator. The manufacturer employs internal, validated positivity thresholds for each target pathogen; raw read counts above these thresholds are reported as "detected." In the present case, negative results for specific microorganisms (e.g., viruses, fungi, parasites) indicate that no sequence reads attributable to these organism groups met the assay-specific detection threshold. Results for microorganisms that are recognized components of the normal respiratory microbiota (e.g., *Streptococcus mitis*) were interpreted with caution and in the context of the patient's clinical presentation.

The following methodological limitations should be noted: (i) tNGS, like all amplification- based methods, is subject to amplification bias and may not detect organisms below the assay's limit of detection; (ii) tNGS cannot definitively	distinguish between viable	organisms, colonizing organisms, and active infection—clinical correlation is essential; (iii) the detection of antimicrobial resistance is based on genotype and may not always correlate with phenotypic resistance; (iv) a negative result does not completely exclude the presence of a pathogen, particularly if the organism is present at very low titers or if its genomic sequence is not adequately represented in the reference database; and (v) specific technical parameters including per-pathogen detection thresholds, unique

non-duplicate read counts, and normalized abundances (e.g., RPKM) were not provided in the standard clinical report generated by the	commercial	assay and are retained as proprietary information by the manufacturer.

**References**

1.	Society of Clinical Microbiology, China International Exchange and Promotion Association for Medical and Healthcare. Expert consensus on the application and practice of targeted next-generation sequencing in infectious diseases [Article in Chinese]. Natl Med J China. 2024;104(48):4375–4383. doi:10.3760/cma.j.cn112137-20240927-02208
